# Psychophysiological Responses to Salsa Dance

**DOI:** 10.1371/journal.pone.0121465

**Published:** 2015-04-10

**Authors:** Laura Guidetti, Cosme Franklim Buzzachera, Gian Pietro Emerenziani, Marco Meucci, Francisco Saavedra, Maria Chiara Gallotta, Carlo Baldari

**Affiliations:** 1 Department of Movement, Human, and Health Sciences, University of Rome Foro Italico, Rome, Italy; 2 Department of Physical Education, North University of Parana, Londrina, Brazil; 3 Department of Health and Exercise Science, Appalachian State University, Boone, North Carolina, United States of America; 4 Research Center for Sport, Health, and Human Development, University of Tras-Os-Monte and Alto Douro, Vila Real, Portugal; Texas A&M University, UNITED STATES

## Abstract

Speculation exists whether dance provides physiological stimuli adequate to promote health and fitness benefits. Unfortunately, research to date has not addressed the affective and exertional responses to dance. These responses are of interest as positive affective and exertional responses experienced during physical activity may play an important role in predicting adherence. The present study aims to examine the psychophysiological responses of different Salsa dance styles. Ten pairs of dancers performed two different structured lessons of Salsa dance, including *Typical Salsa* and *Rueda de Casino* lessons, and a non-structured Salsa dance at a night club. Physiological responses (i.e., percent of heart rate reserve; %HRR) were continuously assessed and perceived exertion and affective valence were rated every 15 min throughout the trials. %HRR responses differed between the Salsa dance styles (%HRR from 41.3 to 51.9%), and participants were dancing at intensities near their ventilatory threshold. Specifically, *Typical Salsa* lesson elicited lower %HRR responses than *Rueda de Casino* lesson (*p* < 0.05), but similar %HRR responses to Salsa dance at a night club condition (*p* > 0.05). Surprisingly, exertional (from 8 to 11) and affective (from +3 to +5) responses were unaffected by Salsa dance styles (*p* > 0.05). These data support that different Salsa dance styles provide physiological stimuli adequate to promote health and fitness benefits, and perhaps more importantly, produce pleasurable experiences, which in turn might lead to an increase in adherence to Salsa dancing which likely provides exercise-like health benefits.

## Introduction

It is well known that regular physical activity promotes beneficial effects on health and fitness [1]. Unfortunately, most people do not meet the minimum recommended amounts of physical activity participation [2]. Dropout is considered one of the major contributing factors to the low rates of physical activity participation [3,4]. A study conducted by Dishman and Buckworth [4] estimated that approximately 50% of novice exercisers drop out of physical activity programs within the first six months. Thus, many people withdraw from physical activity programs before physiological gains occur. Identification of potential factors affecting adoption and maintenance of physical activity have been considered one of the greatest challenges for exercise researchers [5].

The amount of pleasure one experiences during exercise might play a significant role in later physical activity participation [6,7]. A positive affective response experienced during exercise might lead to a greater enjoyment of the activity, create a positive memory of the exercise experience, and possibly lead to an increased motivation for future physical activity behaviour [8–10]. This hypothesis has been presented using the “hedonic” theory of motivation [11], which argues that if people derive pleasure from their physical activity participation, they would seek to repeat this activity. Conversely, if people derive displeasure from their physical activity, the chances of them repeating this activity would be reduced. In a recent study, Williams et al. [7] showed that affective responses to a moderately intense exercise were predictive of physical activity participation up to 6 and 12 months later. However, this relation of affective responses with later physical activity participation became nonsignificant when controlling for perceived exertion, thus suggesting a critical role of both affective and exertional responses to exercise for adherence in physical activity programs.

It is reported that dance provides physiological stimuli adequate to promote beneficial effects on health and fitness (i.e., an intensity between 55/65-90% of maximal heart rate, HR_max_, or between 40/50-85% of oxygen consumption reserve, $\dot{V}$O_2_R, or heart rate reserve, HRR; [12]) [13–15]. Preliminary reports suggest that Salsa dance, which was developed in the late 1960’s, by Cuban and Puerto Rican immigrants in the New York City area and became one of the most practiced Caribbean dances around the world, may result in improvements in fitness and health [16,17]. Indeed, a recent study by Di Blasio and co-workers [16] confirmed that the physiological response to a typical lesson of Salsa dance is adequate to promote health and fitness benefits (53.2% and 60.6%HRR, respectively) in beginner and expert dancers. However, it should be noted that there are currently many recognized styles of Salsa dance, which could lead to different physiological requirements [18]. Some of the differences between dance styles include the timing of steps, particular movements on the dance floor, dancer preference of turns and moves, attitude, dress code, and others. For example, a particular style of Salsa dance the *Rueda de Casino*, which consists of couples dancing together while building a large circle and synchronising their individual figure rotations with those of the group. Each individual takes turns dancing with each member of the circle until the end of the song. A male caller announces the changes in turns, dance patterns, and figures. With this style, many moves have hand signs to complement the calls. This is useful in noisy venues, where spoken calls might not be easily heard.

Interestingly, none of the abovementioned studies provided any information concerning exertional and affective responses to dance, which in turn might play a significant role in determining adherence to that activity [7]. The present study was designed to fill this void. In particular, this study will examine not only physiological, but also exertional and affective responses to three Salsa dance conditions, including a *Rueda de Casino* lesson, a *Typical Salsa* lesson, and Salsa dancing at a night club. Due to the relative paucity of research examining psychophysiological responses to Salsa dance, no directional hypotheses were asserted. In addition, to further verify the relationship between affective responses and physical activity adherence, follow-up data about Salsa dance participation were acquired two years after the experimental trials. It was hypothesized that affective responses would be positively associated with Salsa dance practice at two years follow-up in Salsa dancers.

## Methods

### Participants

Ten pairs of Salsa dancers volunteered to participate in the present study, which was approved by the University of Rome’ Ethics Committee and conducted in accordance with ethical principles stated in the Declaration of Helsinki. The descriptive characteristics of the participants are summarized in Table 1. Written informed consent was obtained from all participants, and the experimental procedures, possible risks, and benefits of the study were explained. All individuals were in good health and were not taking medication known to influence metabolic or cognitive functions. All participants were classified as intermediate level and they took Caribbean dance lessons on average for 4 hr. per week over a period of at least two years.

**Table 1:** Anthropometric characteristics and physiological, exertional, and affective responses to maximal graded exercise test of the participants.

	**Men (*N* = 10)**		**Women (*N* = 10)**	
	**Mean**	**SD**	**Mean**	**SD**
Age (years)	36.3	7.7	38.1	6.1
Body mass (kg)	76.6	10.9	59.9	15.7 *
Stature (cm)	173.2	7.6	162.9	4.6 *
BMI (kg · m^-2^)	25.4	2.8	22.5	5.3
Body fat (%)	15.4	6.6	24.2	*
$\dot{V}{\text{O}}_{2\text{max}}$ (mL · kg.^-1^ · min.^-1^)	39.5	4.7	33.3	5.6 *
$\dot{V}{\text{O}}_{2\text{VT}}$ (mL · kg.^-1^ · min.^-1^)	26.3	4.0	15.3	3.5 **
HR_max_ (beats · min^-1^)	183.7	7.7	182.7	6.1
HR_VT_ (beats · min^-1^)	136.0	14.2	128.3	14.1 * *
RPE_VT_ (6–20)	12.5	1.7	12.6	1.7
Affect_VT_ (-5–+5)	1.30	1.56	1.40	1.71

BMI: body mass index; $\dot{V}{\text{O}}_{2\text{max}}$: maximal oxygen consumption; $\dot{V}{\text{O}}_{2\text{VT}}$: oxygen consumption at-VT; HR_max_: maximal heart rate; HR_VT_: heart rate at-VT; $\dot{V}\text{E}$, maximal pulmonary ventilation; RPE_VT_: rating of perceived exertion at-VT; VT: ventilatory threshold. Significant difference

* *p* < 0.05

** *p* < 0.01.

### Study Design

Each participant completed one orientation session and three experimental trials, scheduled on different days (48 to 72 hr. between trials). On Day 1, individuals underwent an initial screening, anthropometric measurement, and a maximal exercise test. On Days 2 and 3, participants performed two different structured lessons of Salsa dance, namely *Typical Salsa* lesson and *Rueda de Casino* Lesson, respectively. Finally, on Day 4, individuals were allowed to freely dance salsa at a night club without restrictions. A more thorough explanation of each condition is provided in the section *Experimental Trial*. To avoid circadian variations, all trials were conducted between 19:00 and 21:00 hrs. All participants were instructed to refrain from exercise and to avoid alcoholic or caffeinated products in the 24 hr. preceding the tests. They were also instructed to wear the same type of Salsa dance clothing and shoes for the orientation and experimental trials.

### Anthropometric Measurements

Stature (cm.) and body mass (kg.) were measured according to the techniques described by Gordon et al. [19]. Body mass index (kg m^-2^) was calculated as body mass divided by stature squared. Skinfold thickness measurements were taken with a Harpenden caliper (British Indicators^TM^, St. Albans, UK) to the nearest 0.2 mm at the following sites: chest, abdominal, suprailiac, triceps, and thigh. All of these skinfold thickness measurements were taken in triplicate on the right side of the body, and the average values were used. Body density (g cm^-3^) was then estimated from the equations of Jackson and Pollock [20] and Jackson, Pollock, and Ward [21], and then converted to percent of body fat. All anthropometric measures were performed by the same investigator.

### Maximal Exercise Test

The maximal oxygen consumption ($\dot{V}$O_2max_) of the participants was estimated by means of a continuous, maximal graded exercise test on a step. After 5-min. of seated rest, participants began to step up and down on a bench 20 cm high for three minutes each stage in a maximum of eight stages. The up and down step rates for each stage were 10, 15, 20, 24, 28, 31.5, 34.5 and 38 steps per minute. Step rates were chosen to provide increments in oxygen consumption ($\dot{V}$O_2_) of about 5.0 mL kg^-1^ min^-1^ each stage, which was calculated based on the nomogram developed by Margaria et al. [22]. A metronome was set so that each beat corresponded to a single foot movement, and thus one complete up and down step action was represented by four foot movements. To achieve a $\dot{V}$O_2max_ required participants to meet two of the following criteria: (a) a plateau in $\dot{V}$O_2_ (changes of < 150 mL min.^-1^ in the last three consecutive 15-sec. averages), (b) a respiratory exchange ratio of ≥ 1.15, and (c) a heart rate (HR) within 10 beats min.^-1^ of the age-predicted maximum. Therefore, $\dot{V}$O_2max_ was defined as the highest value of $\dot{V}$O_2_ attained after reaching the standard criteria. HR_max_ was defined as the highest HR value recorded over any continuous 30-sec. interval during exercise, whereas HR_Rest_ was considered the average value of HR recorded over the last 2 min. of seated rest [23]. Ventilatory threshold (VT) was estimated offline for each participant by plotting the ventilatory equivalent ($\dot{V}$E/$\dot{V}$O_2_) as a function of $\dot{V}$O_2_ in order to identify the point during exercise where this curve has its minimum value. The level of $\dot{V}$O_2_ at which the lowest value of the ratio $\dot{V}$E/$\dot{V}$O_2_ was found in the individual plot was defined as individual VT [24].

HR (beats min.^-1^) was continuously recorded before and throughout the trial using a HR monitor (RS 400, Polar Electro^**TM**^, Kempele, Finland). $\dot{V}$O_2_, carbon dioxide production ($\dot{V}$CO_2_), and pulmonary ventilation ($\dot{V}$E) were measured on a breath-by-breath basis by a portable gas analysis system (K4 b^2^ Cosmed^TM^, Rome, Italy). The system was calibrated using room air (20.93% O_2_, 0.03% CO_2_) and a certified gas mixture (16% O_2_, 5% CO_2_; Scott Medical Products^TM^, Plumsteadville, USA) prior to each test. In addition, the turbine flowmeter was periodically calibrated using a 3-L syringe according to the manufacturer’s instructions.

### Experimental Trials

All participants performed the three experimental trials in the same order. The first two trials comprised a *Typical Salsa* lesson and a *Rueda de Casino* lesson, respectively. Both lessons were structured as follows: 15 min. of basic Caribbean dance steps (*pasitos*) as warm up, 15 min. of learning a new dance form, and 30 min. of dancing with a partner (for *Typical Salsa* lesson condition) or dancing in a circle with a frequent exchange of partners (for *Rueda de Casino* lesson condition). The first two conditions were structured while the third condition was not structured. The third trial consisted of 60 min. of Salsa dancing at a night club. The participants were dancing in a traditional night club and were allowed to dance freely without restrictions, including swapping partners with each dance. However, they were instructed to complete the 60 min. of Salsa dancing at the same time. The participants were also allowed to drink water, but not alcoholic or caffeinated products, during this trial. Music tempo was set between 40–50 beats per minute corresponding to step cadence of 160–200 beats per minute throughout the trials. The average temperature and humidity were 19.3 ± 1.5°C and 50 ± 8%, respectively, throughout the trials, in a room with a safe and appropriate surface designed for dance and with a space of 1000m^2^ for *Typical Salsa* lesson and *Rueda de Casino* lesson and 1500m^2^ for Salsa dancing at a night club.

HR was continuously recorded before and throughout the trials using a short-range radio telemetry HR monitor (Polar Team System, Polar Electro^TM^, Kempele, Finland). These HR responses were then averaged for the final 30-sec. intervals of every 15 min. period of each trial, as well as 1 min. before exercise. For each participant, heart rate reserve (HRR) was calculated by subtracting HR_Rest_ value from the respective maximal value (i.e., HR_max_). Accordingly, for each 15 min. interval of each of the trials, the increment above resting for each value was divided by the calculated reserve and multiplied by 100 to derive %HRR [23].

### Measures of Perceived Exertion and Affect

Perceived exertion was defined as the subjective intensity of effort, strain, discomfort, and/or fatigue that was felt during exercise [25]. The Borg 6–20 Ratings of Perceived Exertion (RPE) scale [26] was used to assess whole-body perceived exertion during exercise. This scale is a 15-point single-item measure ranging from 6 to 20, with anchors ranging from “no exertion at all” to “maximal exertion”. Perceived exertion was assessed during the last 15 sec. of each 3-min. stage of the maximal exercise test and every 15 min. interval of the experimental trials. The RPE obtained in the maximal exercise test stage corresponding to VT was defined as the RPE_VT_. The Ratings of Perceived Exertion measured by Borg 6–20 scale show mean validity coefficients of 0.62 for HR, 0.63 for $\dot{V}$O_2_, 0.64 for %$\dot{V}$O_2max_, 0.61 for $\dot{V}$E, 0.72 for respiratory rate, and 0.57 for blood lactate [27].

The Feeling Scale (FS) [28] was used to measure “basic” or “core” affective valence (pleasure–displeasure). Commonly used in the assessment of affective responses during exercise [8,10], the FS is an 11-point single-item bipolar measure ranging from +5 to -5, with verbal descriptors of very good (+5), good (+3), fairly good (+1), neutral (0), fairly bad (-1), bad (-3), and very bad (-5). According to Van Landuyt et al. [29], the FS correlates from 0.51 to 0.88 with the valence scale of the Self-Assessment Manikin [30] and from 0.41 to 0.59 with the valence scale of the Affect Grid [31]. In addition, Rose and Parfitt [10] supported the FS as a reliable measure of affective valence, with coefficients of variation ranging from 5% to 11% for $\dot{V}$O_2_ and HR across four exercise bouts. Like the RPE scale, the FS was assessed during the last 15 sec. of each 3 min. stage of the maximal exercise test and every 15 min. interval of the experimental trials. The FS was also measured 1 min. before dance. The affective valence obtained in the maximal exercise test stage corresponding to VT was defined as the affect_VT_.

Participants were anchored to both scales using memory anchoring procedures [8,25]. Separate instructional sets for both RPE and FS were read immediately before the maximal graded exercise test and experimental trials. After reading the instructions, each participants was asked five questions regarding the use of the scales. The purpose of this was to verify that each participant had read and understood the instructions. Unfortunately, the scales were not in view of the participants at all times of the experimental trials. However, the use of memory anchoring procedures and standard instructions before the experimental trials might have attenuated this methodological constraint. Importantly, the order of scale presentation was counterbalanced, but not random, so that the order of FS and RPE scale varied across the 15 min. intervals of the trials in order to minimize any effect of order presentation. However, the order that perceived exertion and affective valence were estimated at a specific time (e.g., 15-min.) was the same for all participants. It should be noted that the participants in this study were allowed to stop dancing to report the exertional and affective estimates, which were recorded by trained technicians.

### Statistical analysis

Data are shown as mean (*M*) ± standard deviation (*SD*). Independent *t*-tests were used to examine gender differences in anthropometric characteristics and physiological, exertional, and affective responses during maximal graded exercise test (*p* < 0.05). A series of two-factor, Salsa dance condition (*Typical Salsa* lesson, *Rueda de Casino* lesson, and Salsa dance at a night club) × time (rest, 15-min., 30-min., and 60-min.) repeated measures analysis of variance (ANOVA) was conducted on %HRR, perceived exertion, and affective valence. Initially, gender was also included as a between-subject factor in all analysis conducted on the dependent variables (i.e., %HRR, perceived exertion, and affective valence). However, none of the main or interaction (e.g., gender × Salsa dance condition, gender × time, and gender × Salsa dance condition × time) effects of gender had a statistical significance, and as a consequence, gender was omitted. Wherever the sphericity assumption was violated in the ANOVA models, degrees of freedom were adjusted and reported using the Greenhouse-Geisser epsilon correction. Partial eta squared (η^2^_p_) was used to determine the size of the effects. For each ANOVA model with a significant Salsa dance condition × time interaction, the simple effects of time were further analysed within each Salsa dance condition. Significant simple effects of time were followed by planned contrasts in which 30-min. and 60-min. values were compared with their 15-min. values, except for affective valence, in which 15-min., 30-min., and 60-min. values were compared with their min. 0 (i.e., pre-exercise) values. Because these comparisons increase the risk for type I error (i.e., to reject the null hypothesis when it should not be rejected), the *p* value for *post hoc* analyses was adjusted according to the Bonferroni correction to 0.05/2 = 0.025 (for affective valence, 0.05/3 = 0.016). Additionally, a linear regression analysis was conducted to further examine the role of affective responses in determining long term Salsa dance participation. For this analysis, self-reported Salsa dance practice (in hours week^-1^) two years after the initial experimental procedures, assessed by face-to-face interviews (*N* = 20), was designed as dependent variable, whereas mean affective responses at 60-min. (e.g., after 30—min. of dancing with a partner) to *Typical Salsa* lesson were entered as predictors. The Typical Salsa lesson was chosen because some of the inherent characteristics, such as learning a new dance form or dancing with a partner, may adequately reflect the reality for most novice and expert Salsa dancers. All data were analysed using SPSS 17.0 for Windows (SPSS, Inc., Chicago, USA).

## Results

There were no gender differences in age, BMI, HR_max_, RPE_VT_ and affect_VT_ (Table 1). However, men were significantly heavier and taller (*p* < 0.05) and had a lower percent of body fat than women (*p* < 0.05). $\dot{V}$O_2max_, $\dot{V}$O_2VT_, and HR_VT_ were significantly higher in men than in women (*p* < 0.05).

**Fig 1:**
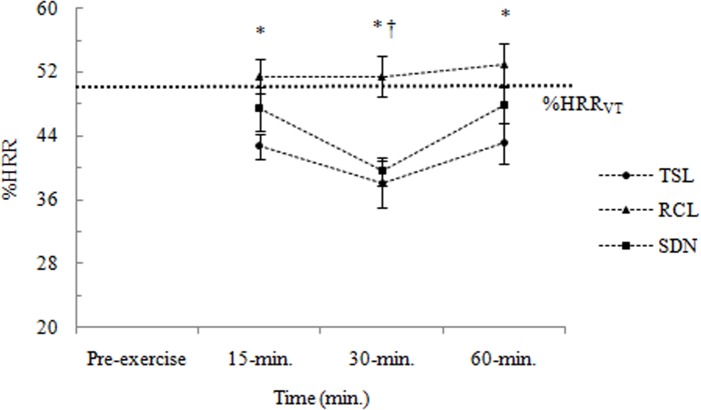
Physiological responses (i.e., %HRR) during the three Salsa dance conditions. Data are shown as means ± SE. TSL: *Typical Salsa* lesson; RCL: *Rueda de Casino* lesson; SDN: Salsa dance at a night club; %HRR: percent of heart rate reserve. %HRR_VT_: percent of heart rate reserve at-VT; VT: ventilatory threshold. * TSL significantly different from SDN; † TSL significantly different from RCL.

%HRR responses to three different Salsa dance conditions are shown in Fig. 1. There were significant main effects of Salsa dance condition *F*(2,38) = 15.736, *p* < 0.05, η^2^_p_ = 0.453, and time *F*(2,38) = 9.285, *p* < 0.05, η^2^_p_ = 0.328. However, there was not a significant interaction between these factors *F*(4,76) = 1.177, *p* > 0.05, η^2^_p_ = 0.058. Repeated planned comparisons found that %HRR responses were significantly higher at 15-min. compared with 30-min. (*p* < 0.025), but were similar to the 60-min., regardless of Salsa dance condition. These analyses also revealed that *Typical Salsa* lesson elicited lower %HRR responses than *Rueda de Casino* lesson, but similar %HRR responses to Salsa dance at a night club condition (except for 15-min.).

**Fig 2:**
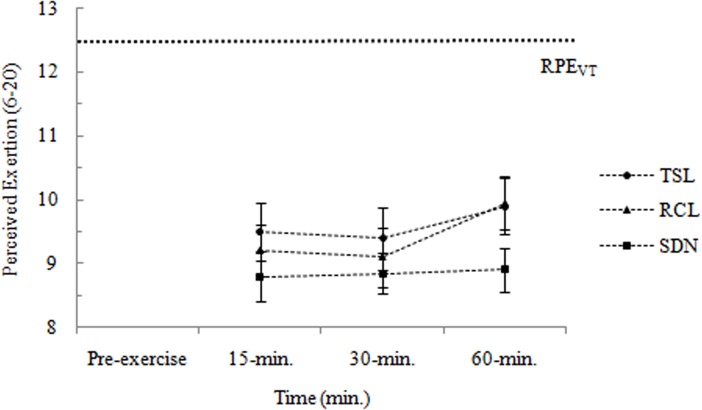
Exertional responses (6–20) during the three Salsa dance conditions. Data are shown as means ± SE. TSL: *Typical Salsa* lesson; RCL: *Rueda de Casino* lesson; SDN: Salsa dance at a night club; RPE_VT_: rating of perceived exertion at-VT; VT: ventilatory threshold.

Exertional responses to three different Salsa dance conditions are shown in Fig. 2. There were no significant main effects of Salsa dance condition *F*(2,38) = 2.297, *p* > 0.05, η^2^_p_ = 0.108 and time *F*(1.539,29.247) = 1.992, *p* > 0.05, η^2^_p_ = 0.095. Similarly, there was not a significant interaction between these two factors *F*(2.725,51.776) = 0.343, *p* > 0.05, η^2^_p_ = 0.018. Repeated planned comparisons found that there were no significant changes in perceived exertion (in comparison with 15-min. values) at 30-min. and 60-min. for the three different Salsa dance conditions (*p* > 0.025). These analyses also revealed that exertional responses did not differ between the three Salsa dance conditions.

**Fig 3:**
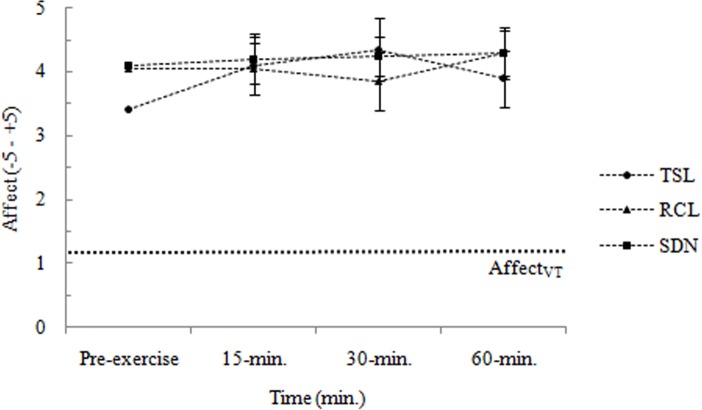
Affective responses (-5–+5) during the three Salsa dance conditions. Data are shown as means ± SE. TSL: *Typical Salsa* lesson; RCL: *Rueda de Casino* lesson; SDN: Salsa dance at a night club; Affect_VT_: affect at-VT; VT: ventilatory threshold.

Affective responses to three different Salsa dance conditions are shown in Fig. 3. There were no significant main effects of Salsa dance condition *F*(1.458,27.693) = 0.653, *p* > 0.05, η^2^_p_ = 0.033, and time *F*(3,57) = 2.133, *p* > 0.05, η^2^_p_ = 0.101. However, there was a significant interaction between these two factors *F*(3.295,62.602) = 3.86, *p* < 0.05, η^2^_p_ = 0.144. This indicates that the changes in the affective responses during the three Salsa dance conditions differed as a function of time. Therefore, the interaction was deconstructed into the simple effects of time within each Salsa dance condition. For the *Typical Salsa* lesson, the effect of time was significant *F*(3,57) = 3.853, *p* < 0.016, η^2^_p_ = 0.169. Planned contrasts found that affective responses were significantly lower at min. 0 (i.e., pre-exercise) compared with 30-min., but were similar to the 15-min. and 60-min. In contrast, there were no significant effects of time for *Rueda de Casino* lesson *F*(1.924,36.557) = 1.567, *p* > 0.016, η^2^_p_ = 0.076, and Salsa dance at a night club *F*(2.162,41.089) = 0.704, *p* > 0.016, η^2^_p_ = 0.036.

Following two years the initial experimental procedures of this study, weekly Salsa dance practice was maintained or improved: 4.6 ± 1.2 hours week^-1^ and 6.4 ± 1.7 hours week^-1^ for women and men dancers, respectively, in comparison with the initial mean value of 4 hours week^-1^ for the 10 studied pairs. Linear regression analysis were separately conducted for men and women dancers, with mean affective responses to *Typical Salsa* lesson as predictors and Salsa dance practice two years after the initial experimental procedures as criteria. Mean affective responses at 60-min. to the *Typical Salsa* lesson were positively associated with Salsa dance practice at 2 years follow-up (*p* < 0.05), with betas of 0.66 (R^2^ = 0.44) and 0.69 (R^2^ = 0.48) for women and men dancers, respectively.

## Discussion

The purpose of this study was to examine whether psychophysiological responses of non-professional Salsa dancers are influenced by different Salsa dance conditions. To this end, the present study design was comprised of participants who performed three experimental trials (i.e., *Typical Salsa* lesson, *Rueda de Casino* lesson, and Salsa dance at a night club) on separate days. The results suggested that a *Typical Salsa* lesson elicits lower %HRR response than *Rueda de Casino* lesson, but similar %HRR response to Salsa dance in a night club. Interestingly, neither exertional nor affective responses differed between the three Salsa dance conditions. Therefore, the main findings of the present study demonstrate that physiological responses, but not exertional and affective responses, are influenced by the Salsa dance condition.

As noted by many authors [13–15], dance is considered one of the most common and practiced forms of physical activity. In particular, Caribbean dances have been widely practiced by many people over the years in order to promote benefits for physical and mental health. In a recent study, Di Blasio and co-workers [16] confirmed that a typical lesson of Salsa dance provides a physiological stimulus sufficient to promote benefits for fitness and health. By using a sample of expert and novice Salsa dancers, those authors found that participants perform moderate-intensity activity during a typical Salsa lesson, corresponding to 53.2% and 60.6%HRR for experts and novices, respectively. These results are consistent with the findings of the present study, which demonstrate that the physiological responses to Salsa dance were within the exercise intensity range recommended by the American College of Sports Medicine [12] to promote fitness and health benefits, regardless of Salsa dance condition (41.3%, 51.9%, and 45.0%HRR for *Typical Salsa* lesson, *Rueda de Casino* lesson, and Salsa dance at a night club, respectively). Specifically, the *Rueda de Casino* lesson elicited higher %HRR responses than the *Typical Salsa* lesson and Salsa dance at a night club. The reasons for this phenomenon are unclear; however, a plausible explanation might be related, at least in part, to partners and recovery time. A *Rueda de Casino* lesson is characterized by more frequent exchanges of partners and reduced intervals of time during which they could recover; otherwise, during a *Typical Salsa* lesson, participants do not change their partners frequently, and this condition is characterized by a long recovery time in order to learn new Salsa figures. This long recovery time might be a potential reason for the lower %HRR responses at 30-min. compared with 15-min. and 60-min. (Fig. 1). Interestingly, these %HRR responses were unaffected by gender. This seems abnormal, as males are the leaders, and therefore have to use their arm and core muscles much more than do the females. The small sample size in this study might have contributed, at least in part, to these findings. Taken together, the results confirmed that Salsa dance provide physiological stimuli adequate to promote health and fitness benefits; however, dance professionals should take into account the differences of each Salsa dance style.

It has been proposed that when examining the relationship between exercise intensity and affective responses, exercise intensity should be defined in relation to ventilatory threshold to ensure a physiological equivalence between individuals [8]. When compared with the HR values recorded at VT during the maximal graded exercise test, HR values calculated from the three Salsa dance conditions revealed that participants elicit %HRR responses slightly below or around to their VT. These findings are similar to prior reports using dance as form of physical activity [13,15] and perhaps more importantly, are also consistent with the fundamental assumptions of the dual mode model [8], which suggest an interplay of cognitive appraisal processes and interoceptive cues in the generation of affective responses during exercise. According to this model, cognitive appraisal processes are the primary determinants of affective responses as the exercise intensity is below or near to the VT. At these exercise intensities, affective responses are considered pleasant by most people but also unpleasant by many other people. Once the exercise intensity is beyond the VT, interoceptive cues gain salience and become the primary determinant of affective responses. At exercise intensities exceeding the VT, affective responses tend to be homogeneously negative. In this context, it is surprising that participants reported stable, homogeneous positive affective responses during the three Salsa dance conditions. In particular, they reported affective responses, on average, varying from +3 (i.e., “good”) to +5 (i.e., “very good”), irrespective of Salsa dance condition. Moreover, only a small number of participants reported decreases in affective responses from baseline to end of the activity (20%, 10%, and 10% of participants for the *Typical Salsa* lesson, *Rueda de Casino* lesson, and Salsa dance at a night club conditions, respectively). This positivity of the affective responses was not affected by considerable differences in %HRR responses to the Salsa dance conditions. This suggests that these affective responses might have been related, in part, by a number of factors such as exercise experience, perception of ability, attitudes, social interaction, intentions, dissociative factors like music, or even participants’ desire to give socially desirable answers [9,32]. Nevertheless, positive affective responses generated by exercise may lead to greater enjoyment of the exercise, promote a positive memory of that activity, and consequently, contribute to maintain or increase the motivation for future physical activity behaviour [7,9]. Indeed, the mean affective responses to the *Typical Salsa* lesson were positively associated with the Salsa dance practice (in hours week^-1^) two years later. However, caution should be taken when interpreting the Salsa dance adherence results. The influence of self-reported data from a face-to-face interview, socially desirable answers and other potential explanations for the relation of affective valence with Salsa dance practice should be subject of future investigations.

Although the affective responses experienced during physical activity may play a role in predicting adherence to physical activity programs, the exertional responses also have consequences for future physical activity behaviour [33]. A recent study by Williams et al. [7] found that affective responses to exercise predicted self-reported physical activity 6 and 12 months later. However, this relationship between affective responses and future physical activity participation became nonsignificant after controlling for ratings of perceived exertion. Therefore, the assessment of both affective (“how” a person feels) and exertional (“what” a person feels) responses should be made for a more complete description of subjective experience to exercise [28] and prediction of a reduced later physical activity participation. In the present study, participants reported perceived exertion values, on average, varying from 8 (i.e., between “extremely light” and “very light”) to 11 (i.e., “light”) on the 6–20 Borg RPE scale, irrespective of Salsa dance condition. This similarity in the exertional responses may be surprisingly, given the principles of Borg’s model of effort continua [26] argues that, as physiological requirements increases, there is a linear increase in perceived exertion. The reasons for this finding are unclear but the influence of positive cognitive-social factors and dissociative cues to Salsa dance might be plausible. Future studies are encouraged to explore how specific cognitive-social factors and associative/dissociative cues affect exertional and affective responses to dance.

Some strengths and limitations within the present study warrant mention. The most prominent strength was the assessment of not only physiological responses, but also exertional and affective responses to Salsa dance. The use of repeatedly assessed affective responses before and during the Salsa dance lessons may be another strength of this study since few studies have embraced this methodological approach [8]. However, prior studies suggested that affective responses should be assessed not only before and during exercise, but also immediately following exercise [34] in order to provide a more complete temporal description of affective responses. Thus, in this study, the lack of assessment of affective responses after exercise may be acknowledged as a limitation. In addition, the present study did not approach the assessment of affect through the circumplex model of Russell and colleagues [35], which incorporates affective valence and activation as orthogonal and bipolar dimensions. The use of both dimensions may provide a more complete examination of the affective experience during exercise [8]. However, this study did not include the measurement of activation since the activation dimension of the circumplex model was not central to the purposes of this investigation. Finally, the participation of experienced, but non-professional Salsa dancers might have limited generalizability to dancers from different expertise levels. Some suggest that beginner dancers might also have more pleasant and tolerable experiences to Salsa dance (16), which in turn might lead to an increase in Salsa dance adherence. A more consistent Salsa dance adherence would likely promote improvements in health and fitness. However, because no study has examined the psychophysiological responses to Salsa dance and its relation with adherence in beginner dancers, this hypothesis is more exploratory in nature.

In summary, the current findings reveal that physiological responses of non-professional Salsa dancers are influenced by the Salsa dance condition. Conversely, neither exertional nor affective responses are affected by different Salsa dance conditions. These data indicate that subtle %HRR differences to Salsa dance might not be sufficient for increasing perceived exertion or reducing affective feelings of habitual Salsa dancers. Further studies are warranted to examine the positive impact of cognitive-social factors and associative/dissociative cues on exertional and affective responses to dance. From an adherence standpoint, the positivity of both exertional and affective responses generated by Salsa dance would be important for leading individuals to a greater enjoyment of the activity, promote a positive memory, and consequently, preserve the increased motivation for future dance behaviour. Finally, from a public health perspective, this research has shown that Salsa dancing could be a useful strategy for promoting regular physical activity in primary prevention programs.
